# Exploring the interplay of mental health knowledge, stigma, and social distance among clinical nurses: a study in Liaoning, China

**DOI:** 10.3389/fpsyt.2025.1478690

**Published:** 2025-02-28

**Authors:** Weiwei Wang, Junhong Xia, Wei Chen, Junhua Ye, Kun Xie, Zhuona Zhang, Siti Mardhiana Binti Mohamad, Ahmad Naqib Shuid

**Affiliations:** ^1^ Medical College, Nanchang Institute Technology, Nanchang, China; ^2^ Institut Perubatan dan Pergigian Termaju, Universiti Sains Malaysia, Penang City, Malaysia

**Keywords:** mental health knowledge, stigma, social distance, clinical nurses, interdisciplinary care, nursing environment

## Abstract

**Background:**

Stigma related to mental health conditions has a negative impact on both the nursing staff and their patients. Most of the current research on stigma explores the impact of single factors on stigma and does not explore the relationship between knowledge, social distance and stigma among clinical nurses.

**Methods:**

A convenience sampling method was used to conduct a questionnaire survey among 628 nurses from five hospitals in Liaoning Province in March 2021 and June 2021 using a combination of online and offline methods. To study the negative attitudes toward patients with mental illness among clinical nurses and to analyze the relationship between mental ill health stigma, social distancing, and mental health knowledge among nurses. The questionnaire includes Sociodemographic data, Scale for Assessing the Stigma of Mental Illness in Nursing (score range: 20~100, the higher the score, the less stigma attached to mental ill health), Mental Illness Social Distance Scale (score range: 6~30, the higher the score, the greater the social distance) and Mental Health and Mental Health Knowledge Questionnaire (score range: 0~25, the higher the score, the higher the knowledge level).

**Results:**

The nurses’ mental ill health stigma score was 58.96 ± 9.38 points, the mean scores of psychiatric and general nurses were 58.86 ± 9.33 and 59.41 ± 9.58 points, respectively. Willingness to become a psychiatric nurse is a factor influencing the stigma of mental illness. The stigma of mental ill health, social distance and mental health knowledge of nurses are positively correlated (P<0.01). The mediation analysis demonstrated that mental health stigma significantly mediated the relationship between mental health knowledge and social distance (a= 0.599, P<0.001; b= -0.194, P<0.001). After accounting for stigma, the direct effect of mental health knowledge on social distance was no longer significant (c’=-0.007, P=0.078), highlighting the central role of stigma in this relationship.

**Conclusions:**

Nurses mental ill health stigma is moderate and is the main mediating effect between mental health knowledge and social distancing. The pivotal role of mental health knowledge in shaping nurses’ attitudes and behaviors pertaining to social distancing in the context of mental illness. By combating stigma and enhancing mental health literacy among healthcare practitioners, we can foster environments that promote inclusive and compassionate care practices, thereby ameliorating patient outcomes and redressing disparities in mental health treatment.

## 1 Introduction

Approximately 1 in every 8 people globally, or 970 million individuals, live with a mental ill health, a number that has increased due to greater awareness and improved mental ill health diagnostics (1). According to WMH surveys in 29 countries, it is estimated that approximately one in two people will suffer from at least one mental disorder by the age of 75 (2). The growing number of people with a mental health condition also imposes a significant burden on the economy, projected to rise to $6 trillion per year by 2030 (3). The Global Burden of Disease study highlights that mental ill health have been a significant contributor to the global burden of disease over the past 30 years (4). Low-and middle-income countries account for about three-quarters of the global burden of mental ill health. As a middle-income country, disability-adjusted life years (DALY) due to mental, neurological and substance use disorders in China account for about 15% to 20% of the global total, ranking first in the world (5). Such figures underscore the urgent need for targeted interventions to address the growing public health and socio-economic challenges posed by mental ill health.

The global ramifications of mental health disorders are particularly evident in China, where the convergence of a substantial population and an increasing prevalence of mental health issues poses significant public health and socio-economic challenges. Mental health represents a critical public health concern in China, characterized by a substantial patient population and considerable socio-economic burdens. Studies have indicated that in 2018, around 150 million people in China were affected by various mental ill health, with an incidence rate of approximately 17.5% (6). In 2021, the number of registered people with a severe mental ill health rose to 6.6 million (7). As of 2023, the China Mental Health Survey shows that the lifetime prevalence of mental ill health in China is about 16.6% (8), which means that about 230 million people will suffer from mental ill health in their lifetime. This growing burden places tremendous pressure on healthcare systems, particularly on nurses, who play a frontline role in providing care to people with a mental health condition. However, nursing stigma toward mental ill health remains a significant barrier to effective care. Thornicroft has stated, “In no country, society, or culture do people with a mental health condition receive the same acceptance and recognition as others” (9). The specific manifestations of mental ill health (10) often lead to these individuals being stigmatized as “different.” Nurses may also harbor discriminatory attitudes toward these patients, perceiving them as incompetent, unpredictable, destructive, and violent (11, 12). This discrimination can lead to a decline in care quality, hindering patient recovery and therapeutic outcomes. For clinical nurses, such stigma can negatively impact their professional identity and social interactions (13), posing significant barriers to their involvement in psychiatric nursing (14) and hindering the discipline’s development (15).

Stigmatization affects patients by diminishing their empowerment, social inclusion, quality of life, willingness to seek help, and compliance with treatment (16). It exacerbates clinical symptoms, disrupts treatment, and damages job prospects and social relationships, leading to social isolation and involuntary medical behaviors, imposing heavy physical and mental burdens on patients and their families (17). Goffman (18), a key figure in stigma research, defined stigma as a socially constructed phenomenon where societal norms discredit an individual’s identity. He highlighted its dynamic nature and how stigmatized individuals manage their “spoiled identity.” The most widely accepted perspective today, proposed by Rusch et al. (19) conceptualizes stigma as involving stereotype (cognitive level), prejudice (emotional level), and discrimination (behavioral level). Stigma is socially constructed and manifests as exclusion, blame, or demeaning behaviors due to expected adverse social judgments (20). This social construction affects intellectual, emotional, and behavioral dimensions. Numerous studies have explored the link between knowledge and mental illness stigma, with desired social distance being a key behavioral response indicator. This measure reflects the degree of closeness or distancing that individuals hypothesize or desire in relation to others, serving as a proxy for group attitudes and social behaviors (21). When individuals endorse negative stereotypes about mental ill health, they exhibit prejudice and negative emotional reactions, leading to discrimination and exclusion of the stigmatized group, impacting social interactions. This phenomenon has been observed in China, where mental ill health has historically faced cultural and moral stigma (22). Studies indicate that people maintain greater social distance from stigmatized groups and seldom engage with them, aggravating public stigmatization (23, 24). When stigmatized individuals are aware of societal stigma, it increases social distance, forming a vicious cycle (25). This issue is also present among medical staff, as mental health workers often maintain significant social distance from patients and reject them (26).

Most existing studies focus on public discrimination against people with a mental health condition, with few addressing exclusionary behaviors and social distancing among nurses. According to the “Knowledge-Attitude-Practice model,” knowledge is crucial in reducing in-group bias against out-group members (27). Knowledge is also pivotal in combating mental health-related stigma (28, 29). Improving public mental health literacy can reduce stigmatizing attitudes (30). A systematic review of 515 studies identified knowledge as a key factor in reducing out-group bias (31). However, some studies reveal that while nurses possess good knowledge of mental ill health, their attitudes remain negative (32).

Currently, most stigma studies focus on people with mental health condition or their families, with limited research on nurses stigmatization. Qualitative studies suggest that stigma among nurses stems from attitudinal knowledge and behavior (33). However, quantitative research supporting these findings remains limited, and existing studies have yet to fully explore the nuanced relationship between knowledge, behavior, and stigma. This study aims to examine the correlation among mental health knowledge, social distance, and stigma among nurses, with the goals to (1) investigate the current state of mental ill health stigma among nurses and analyze its influencing factors, and (2) explore the mediating effects of stigma on the relationship between mental health knowledge and social distancing among clinical nurses.

## 2 Materials and methods

### 2.1 Sample

In order to capture different perspectives in the nursing industry and effectively collect data from different levels and regions, this study used convenience sampling to select nurses who volunteered to participate in this study from five hospitals in Jinzhou and Dalian, Liaoning Province. 1) Affiliated Hospital of Jinzhou Medical University; 2) Jinzhou Kangning Hospital; 3) Wafangdian Central Hospital; 4) Wafangdian city women and Children’s Hospital and 5) Wafangdian city fourth hospital between March 2021 and June 2021. A total of 657 questionnaires were distributed, all of which were recovered. Among them, 628 were deemed valid, resulting in an effective recovery rate of 95.59%.

### 2.2 Inclusion and exclusion criteria

Inclusion criteria: 1) possess a nursing qualification certificate 2) volunteers for the study.

Exclusion criteria: 1) currently on vacation 2) Non-clinical frontline work.

### 2.3 Data collection

This study used an online and offline method to collect data. Before distributing the questionnaire, the purpose and significance of the questionnaire were explained to the nurses, and the precautions for filling in the questionnaire were informed. After obtaining the consent of the nurses, the questionnaires were distributed to the nurses on site, and the nurses completed the questionnaires independently. The time for filling in the questionnaire was 5 to 10 minutes. After the questionnaires were completed, they were collected on the spot to check whether there were any omissions or errors. The nurses confirmed and modified them in time to ensure the integrity and quality of the questionnaires. The scales selected in this study have good reliability and validity, which can ensure the accuracy of the measurement. The researcher herself was responsible for the distribution and collection of the questionnaires, ensuring that the questionnaires were filled in within the specified time and independence during the filling process. In the process of collecting the questionnaires, the integrity of the questionnaires was checked, and the missing options were communicated and checked with the research subjects in time to ensure the integrity of the questionnaires. After the questionnaires were collected, the quality of the questionnaires was checked, and the questionnaires with a completion rate of less than 80% were deleted to ensure the scientific nature of the research. When entering the data, two people checked, and if there were any differences, the original data was found in time for verification to ensure the accuracy of the data.

### 2.4 Instruments

#### 2.4.1 Sociodemographic questionnaire

The sociodemographic questionnaire included items on sex, age, education level, monthly income, level of interaction with mental health patients, exposure to mental health education, and willingness to specialize in psychiatric nursing.

#### 2.4.2 Scale for assessing the stigma of mental illness in nursing

The Scale for Assessing the Stigma of Mental Illness in Nursing (SASMIN) was developed in 2020 by Spanish scholar Dr. Meritxell based on the Peplau psychodynamic nursing model (34). A total of 20 items in 3 dimensions are scored on a 5-point Likert scale: 1 is completely disagree, and 5 is completely agree. They included Violence/Dangerousness (8 items), Disability (5 items), Irresponsibility and Lack of Competence (7 items). Items 2, 3, 5, 10, 13 and 15 are scored directly, and the rest are scored in reverse. The higher the score, the less stigma attached to mental ill health. The intragroup correlation coefficient was 0.906, and the Cronbach’s α coefficient was 0.825. In 2021, the Chinese version of the Scale for Assessing the Stigma of Mental Illness in Nursing was developed by myself and my research team (35), and its reliability and validity were tested. The results showed that the ICC value of the total table was 0.760, the retest reliability was 0.839, and the Cronbach’s α was 0.863, indicating good reliability and validity. In this study, the Cronbach’s α coefficient of this scale was 0.856, and the retest reliability was 0.884, indicating good reliability and validity.

#### 2.4.3 Mental health and mental health knowledge questionnaire

The Mental Health and Mental Health Knowledge Questionnaire is one of the questionnaires on the investigation and evaluation of mental health work issued by the China Health and Family Planning Commission (former General Office of the Ministry of Health) in 2010 (36). The content of the questionnaire is basic knowledge of mental health. The higher the total score is, the better the mental health knowledge. According to the National Mental Health Work Plan (2015–2020) (37), the awareness rate of mental health knowledge of the urban general population should reach 70%, that of the rural population should reach 50% and that of school students should reach 80% in 2020.The questionnaire aligns with national mental health education goals.

#### 2.4.4 Social distance scale

American scholar Link (38) compiled a Social Distance Scale for mental illness, which was used to evaluate the degree of public rejection of people with a mental health condition. The theory is that the more biased people are against a social group, the less willing they are to associate with members of that group. The single-dimension, six-item scale includes living in the same community as a mentally ill person, being a neighbor of a mentally ill person, working with a mentally ill person, communicating with a mentally ill person on a daily basis, being a friend of a mentally ill person, and being a spouse of a mentally ill person. The Likert 5-point scoring method is adopted, and the answer of each item is the degree (out of 5) of willingness of the respondent, namely, “very willing, willing, neutral, unwilling, very unwilling”, recorded as 1 to 5 points, respectively. The cumulative score of the 6 items is the total social distance score, and the higher the score is, the greater the social distance between the individual and the external group. In this study, the intragroup correlation coefficient of this scale was 0.901, and the Cronbach’s α coefficient was 0.883, showing good reliability and validity.

### 2.5 Statistical methods

Epidata Entry Mac version 13.0 was used for data entry and data documentation, SPSS for Mac version 26.0 was used for statistical analysis, and Sociodemographic data and stigma assessment scores, social distance scores, and mental health knowledge scores were statistically analyzed using the mean, standard deviation, frequency and component ratio. The Z-score was used to standardize and classify the stigma scores to explain the variability within the sample. T-test was used for comparison between two groups, one-way ANOVA was used for comparison of multiple groups, and multiple linear regression was used for analysis of influencing factors. Pearson’s two-tailed test was used to analyze the relationship between social distance from mental ill health, mental health and mental health knowledge among clinical nurses. To explore the mediating effect of social distance between mental health knowledge and stigma of mental ill health, the process bootstrap method was used, with a=0.05 as the test criterion.

### 2.6 Ethical considerations

Ethical approval will be obtained from the Institutional Review Board (IRB) or relevant ethics committee prior to data collection. Informed consent will be obtained from all participants, and steps will be taken to ensure the confidentiality and anonymity of their responses. Participants will be informed of their right to withdraw from the study at any time without consequences.

## 3 Result

### 3.1 The sociodemographic questionnaire on the stigma of mental ill health in nurses

The study involved 628 nurses, consisting of 589 females (93.79%) and 39 males (7.21%). Regarding age distribution, young adults aged 20 to 39 comprised 77.1%, those aged 40 to 49 comprised 20.4%, and those aged 50 or older made up only 2.5%. The univariate analysis results indicate that, among sociodemographic factors, the willingness to become a psychiatric nurse is a significant factor influencing nurses’ stigma toward mental illness (P<0.0001). Further details can be found in **Table 1**.

**Table 1:** Sociodemographic information of the research subjects (n=628).

Item	N (%)	P	F/t
Sex		0.992	-0.010
Male	39 (6.20)		
Female	589 (93.80)		
Age		0.555	2.547
20~	219 (34.90)		
30~	265 (42.20)		
40~	128 (20.40)		
≥50	16 (2.50)		
Family member with a mental illness		0.765	0.300
Yes	28 (4.50)		
No	600 (95.50)		
Would like to be a psychiatric nurse		0.0001	3.822
Yes	176 (28.00)		
No	452 (72.00)		
Level of education		0.263	1.331
Technical secondary school	56 (8.90)		
Junior College	95 (15.10)		
Undergraduate	452 (72.00)		
Postgraduate	25 (4.00)		
Current job		0.055	2.342
Nurse	139 (22.20)		
Nurse practitioner	243 (38.70)		
Nurse in charge	196 (31.20)		
Deputy Chief nurse	49 (7.80)		
Chief nurse	1 (0.20)		
Taken courses related to mental illness		0.662	0.438
Yes	313 (49.80)		
No	315 (50.20)		
Degree of contact with the mentally ill		0.697	- 0.389
No Contact	189 (30.10)		
Occasionally	316 (50.30)		
Now and then	19 (3.00)		
Frequent	103 (16.40)		
Monthly household income (yuan)		0.212	1.557
<3000	60 (9.60)		
3000~	437 (69.60)		
≥5000	131 (20.90)		
Have you ever heard of stigma		0.274	-1.096
Yes	191 (30.40)		
No	437 (69.60)		
Are you a psychiatric nurse		0.564	0.577
Yes	116 (18.47)		
No	512 (81.53)		

### 3.2 Current status of mental ill health stigma among nurses

The Scale for Assessing the Stigma of Mental Illness in Nursing yielded total scores of 58.96 ± 9.38 points for nurses, 58.86 ± 9.33 points for general nurses, and 59.41 ± 9.58 points for psychiatric nurses. Specifically, the scores for violence and danger were 22.68 ± 5.71 points, for ability deficiency were 14.64 ± 3.54 points, and for irresponsibility and lack of ability were 21.64 ± 3.83 points (refer to **Table 2** for specifics). To evaluate nurses’ levels of mental ill health stigma, the total stigma score for each subject was converted to a Z score. Stigma levels were categorized into four tiers: no stigma, mild stigma, moderate stigma, and severe stigma. With an average Z value of 0 and a general range of -3 to 3, a Z score below -1.5 indicated severe stigma, -1.5 to 0 indicated moderate stigma, 0 to 1.5 indicated mild stigma, and above 1.5 indicated no stigma. The analysis revealed that out of the sample, 69 cases (10.99%) exhibited no stigma, 281 cases (43.47%) showed mild stigma, 248 cases (39.49%) had moderate stigma, and 38 cases (6.05%) displayed severe stigma (refer to **Table 3** for detailed breakdown).

**Table 2:** Mental illness stigma scores of nursing staff.

Item	Score ( $\bar{\text{x}}$ ±*s*)			
	Score (n=628)	Scoring range	General nursers (n=512)	Psychiatric nursers (n=116)
Total score	58.96±9.38	20~100	58.86±9.33	59.41±9.58
Violence/Dangerousness	22.68±5.71	8~40	22.74±5.93	22.40±4.65
Disability	14.64±3.54	5~25	14.60±3.53	15.26±3.55
Irresponsibility and Lack of Competence	21.64±3.83	7~35	21.62±3.73	21.76±4.27

**Table 3:** Mental illness stigma Z scores of nursing staff.

Item	Z score (%)			
	Total cases (n=628)	Scoring range	General nursers (n=512)	Psychiatric nursers (n=116)
Severe stigma	38 (6.05)	<-1.5	30 (5.86)	11 (9.48)
Moderate stigma	248 (39.49)	-1.5~0	209 (40.82)	44 (37.93)
Mild stigma	281 (43.47)	0~1.5	241 (47.07)	55 (47.41)
No stigma	69 (10.99)	>1.5	32 (6.25)	6 (5.17)

### 3.3 Correlation of mental ill health stigma, social distancing and mental health knowledge among nurses

According to Pearson correlation analysis, the mental ill health stigma score of the nursing staff was negatively correlated with social distance (P<0.0001), negatively correlated with mental health knowledge (P<0.0001), and positively correlated with social distance and mental health knowledge (P<0.01). That is, the degree of mental illness stigma of nursing staff is positively correlated with social distance, and the degree of mental illness stigma is negatively correlated with mental health knowledge. All three are correlated; therefore, this information can be used to analyze the mediating effect. For detailed statistics, please refer to **Table 4**.

**Table 4:** Correlation of mental illness stigma, social distancing and mental health knowledge among caregivers.

Item	Stigma of Mental Illness in Nursing				Social distance
	Stigma	Violence/Dangerousness	Disability	Irresponsibility and Lack of Competence	
Social distance	-0.410**	-0.299**	-0.262**	-0.316**	1.00
Mental health literacy	0.246**	0.285**	0.173**	0.116	-0.107**

**At level 0.01 (two-tailed), the correlation was significant.

### 3.4 The mediating effect of the degree of mental ill health stigma among nurses on social distance and mental health knowledge questionnaire

Structural equation modeling (SEM) was used to test complex relationships between multiple variables simultaneously, including direct and indirect effects. Utilizing the theoretical framework of Knowledge-Attitude-Practice Model, a structural equation modeling was constructed, where mental health knowledge served as the independent variable (X), social distance from mental ill health as the dependent variable (Y), and stigma of mental ill health among nurses as the mediating variable (M), while controlling for sex, age, and education level. The findings revealed significant paths: a=0.599 ((P<0.0001)), b=-0.194 ((P<0.0001)), and c’=-0.007 (*P*=0.078). These results underscore the pivotal role of stigma as the primary mediating factor. Moreover, they indicate that mental health knowledge influences social distance from mental ill health predominantly through the mediation of mental ill health stigma. For comprehensive statistics, please refer to **Tables 5**, **6**.

**Table 5:** Model test of nursing staff stigma mediation.

Y	X	β	SE	*t*	95% confidence interval		R^2^	F	*P*
					LLCI	ULCI			
Stigma	Constant	52.960	1.013	52.262	50.970	54.950	0.060	40.216	0.000
	Knowledge	0.599	0.095	6.342	0.414	0.785			0.000
Social distance	Constant	32.494	1.046	31.075	30.441	34.548	0.169	63.321	0.000
	Stigma	-0.007	0.043	-0.161	-0.092	0.078			0.872
	Knowledge	-0.194	0.018	-10.868	-0.229	-0.159			0.000

**Table 6:** Analysis of the mediating effect of mental illness stigma among nursing staff.

Route	a	b	c'	a*b	BootSE	95% Confidence interval		R^2^
						LLCI	ULCI	
Knowledge→stigma→social distance	0.616	-0.188	0.0313	-0.116	0.031	-0.116	-0.074	0.194
Knowledge→violence/dangerousness→social distance	0.017	-0.351	-0.079	-0.006	0.018	-0.042	0.028	0.137
Knowledge→disability→social distance	0.148	-0.299	-0.041	-0.044	0.017	-0.080	-0.015	0.102
Knowledge→irresponsibility and lack of competence→social distance	0.450	-0.225	-0.017	-0.102	0.025	-0.154	-0.057	0.124

## 4 Discussion

### 4.1 Level of mental health knowledge and stigma among clinical nurses

The findings of this study offer valuable insights into the dynamic interrelationships among mental health knowledge, stigma, and social distance within the cohort of clinical nurses in Liaoning, China. This research underscores the pivotal role that mental health knowledge plays in influencing nurses’ attitudes and behaviors toward individuals with mental illnesses. Importantly, the mediating effect of stigma on the relationship between knowledge and social distance highlights the intricate challenges involved in addressing mental health care disparities in clinical practice. By examining these factors within the nursing profession, the study contributes to an expanding body of evidence that underscores the significance of targeted educational interventions and stigma-reduction strategies. These strategies are essential for enhancing mental health care delivery and promoting a more inclusive clinical environment.

Regarding stigma among clinical nurses, findings from this study indicated an average stigma rating of 58.96 ± 9.38 among nursing staff, with 45.54% (286 cases) from the total sample exhibiting moderate to severe stigma. These results align with those of numerous previous studies (39), indicating a prevalent stigmatized attitude among nursing staff toward individuals with mental illness. Surveys of nursing students (40, 41) revealed similarly negative attitudes toward mental illness. Notably, these attitudes were observed to be more negative in comparison to those documented in a stigma survey involving 192 caregivers, as reported by Jin Xinxia (42). This discrepancy may be attributed to variations in the timing of measurement, given that Jin Xinxia’s study focused on clinical nurses at an earlier stage in their careers. Previous research has indicated that age serves as an independent factor influencing the stigma associated with mental illness among nursing professionals, with younger individuals exhibiting more positive attitudes (43, 44).

Subsequent analyses identified variations in attitudes toward mental illness across different demographic groups. The willingness of nurses to pursue a career in psychiatric nursing can significantly influence the prevalence of mental illness stigma among nursing professionals. Nurses who express a readiness to specialize in psychiatric nursing tend to exhibit lower levels of stigma, a finding corroborated by numerous studies. These studies suggest that an interest in psychiatric nursing is often linked to more positive attitudes and reduced stigma toward mental illness. For example, a study examining the stigmatizing attitudes of nurses and nurse managers toward mental illness found that those who actively supported and assisted patients with mental health conditions exhibited lower levels of stigma (45). This suggests that nurses inclined toward psychiatric nursing roles may have a greater capacity for understanding and empathizing with individuals experiencing mental illness, thereby reducing stigma. Moreover, another study investigated the impact of psychiatric nursing courses on students’ perceptions of mental illness and the field of psychiatric nursing. The results indicated that exposure to psychiatric nursing education significantly reduced negative beliefs about mental illness while fostering more positive attitudes toward psychiatric nursing as a profession (46). These findings highlight the crucial role of education in shaping nurses’ attitudes toward mental health, emphasizing the need for targeted training programs to reduce stigma and enhance mental health care. The results of this study showed that individuals who were willing to become psychiatric nurses usually showed lower stigmatization of mental illness, which may be related to their interest in psychiatric nursing and professional education background. This pathway of action is consistent with the hypothesis of this study. Therefore, further strengthening mental health education and professional training, especially targeted courses and practice opportunities for clinical nurses, may be an effective strategy to reduce stigma, improve nursing quality, and enhance patient outcomes.

Notably, psychiatric nurses demonstrate lower levels of mental health stigma compared to their non-psychiatric counterparts (47). This observation is consistent with the results of this study, although there was no statistically significant difference in the level of stigma between general nurses and psychiatric nurses. These results imply that professional background and roles may play a crucial role in shaping mental illness stigma. The frequent exposure of psychiatric nurses to mental illness cases in their professional environment may contribute to this reduced stigma. Existing quantitative research (48) and qualitative studies (49) support the “contact hypothesis.” However, contradictory evidence exists, such as studies indicating that family caregivers may display high levels of mental stigmatization despite their close interactions with patients. This phenomenon could be attributed to the substantial emotional, psychological, and social stress experienced by family caregivers in their caregiving roles, although the specific mechanisms warrant further investigation.

The results of this study underscore the persistent high degree of mental ill health stigma among nurses, emphasizing the urgency to address, adapt, and ameliorate these stigmatizing attitudes to enhance nursing care quality.

### 4.2 The mediating effect of stigma in the relationship between mental health knowledge and social distancing behavior

The results of this cross-sectional investigation illuminate the complex interplay between mental health knowledge and social distancing behaviors within the realm of clinical nursing, placing particular emphasis on the intervening role of stigma associated with mental ill health. Employing mediation model analysis and Pearson correlation, our study unveiled a significant correlation between the degree of mental ill health stigma among nurses and both their mental health knowledge and social distancing behaviors. Notably, diminished levels of stigma among clinical nurses were found to correspond with heightened mental health knowledge and closer interpersonal proximity. These findings are in consonance with prior research (50), which echoes the theoretical framework linking knowledge acquisition to belief formation and consequent behavioral change, underlining the critical importance of promoting health literacy to cultivate affirmative attitudes and conduct (51). The theoretical model of Knowledge-Attitude-Practice Model (KAP) (52) is one of the models for changing human health-related behaviors, used to understand and analyze the behavior change process. It divides the change in human behaviors into three continuous processes: acquiring knowledge, generating beliefs and changing behaviors. Knowledge is the foundation, belief is the driving force, and behavior change is the goal. Through learning of knowledge, people acquire health knowledge and skills, form health beliefs and attitudes, and finally promote the creation of healthy behaviors (53). The model provides a structured approach to analyze the interaction among the three and identify gaps in education and intervention needs.

However, studies have also hinted at a discrepancy, indicating that despite possessing extensive mental health knowledge, nurses may still harbor negative attitudes toward mental ill health—a phenomenon observed across various healthcare professions (32, 54). In recent years, mental ill health has gradually received attention from society. Mental health knowledge is now disseminated through multiple channels that provide basic psychological knowledge, but does not go deep enough to encourage society to readily accept people with a mental health condition.

Furthermore, our investigation underscores the nuanced role of stigma in mediating the relationship between mental health knowledge and social distancing behaviors among nurses. While mental health literacy serves as a cornerstone for fostering empathy and comprehension toward people with a mental health condition (55), the presence of stigma may impede the translation of this knowledge into supportive actions. Our findings underscore stigma’s function as a barrier, obstructing nurses’ full engagement in social distancing practices when tending to people with a mental health conditions. This underscores the imperative of addressing stigma within healthcare settings to cultivate environments conducive to equitable and compassionate care provision.

The implications of our findings for nursing practice are profound. Recognizing the influence of mental health knowledge on social distancing behaviors, healthcare institutions can institute targeted educational interventions (56) aimed at augmenting nurses’ comprehension of mental health issues and mitigating stigma. Equipping nurses with training programs (57) focused on fostering empathy, cultural sensitivity, and effective communication skills (58) can empower them to navigate intricate patient interactions with confidence and professionalism, ultimately elevating the caliber of care dispensed.

### 4.3 Intervention on improving mental health literacy and reduce stigma among clinical nurses

It’s crucial to stress the significance of providing insights into interventions aimed at enhancing mental health literacy and reducing stigma among clinical nurses. This discussion delves into potential avenues for intervention:

Firstly, implementing targeted education (59) and training programs (60) tailored to the specific needs of clinical nurses can significantly bolster their mental health literacy. These programs should encompass various aspects of mental health, including common disorders, symptoms, treatment modalities, and communication strategies. Utilizing methods such as case studies (61), direct patient interaction (62), role-playing exercises, and interactive workshops (63) can foster active learning and skill development among nurses.

Moreover, developing evidence-based practice guidelines specific to mental health care can furnish clinical nurses with clear protocols and best practices for assessing, managing, and supporting individuals with mental health concerns. It’s imperative to regularly update these guidelines to reflect advances in research and clinical practice, ensuring nurses have access to the most current information and recommendations.

Encouraging interdisciplinary collaboration between clinical nurses and mental health professionals is another pivotal strategy (64). Facilitating regular meetings, case consultations, and joint training sessions can promote knowledge sharing, skill exchange, and mutual support, enabling nurses to learn from experts in the field and access specialized resources and expertise (65).

Establishing peer support networks within healthcare organizations can provide clinical nurses with a safe and supportive space to discuss challenges, share experiences, and seek guidance related to mental health care (66). These networks can help reduce feelings of isolation, normalize discussions around mental health, and foster a culture of empathy and understanding among nurses.

Launching targeted anti-stigma campaigns aimed at raising awareness, challenging stereotypes, and promoting positive attitudes toward mental ill health can effectively combat stigma among clinical nurses. Leveraging various mediums such as posters, seminars, social media (67), and workshops can disseminate accurate information, challenge misconceptions, and encourage empathy and compassion toward individuals with mental health conditions.

Creating a supportive organizational culture that prioritizes mental health awareness and inclusivity necessitates strong leadership commitment (68). Hospital administrators and nursing leaders should actively champion initiatives to improve mental health literacy and reduce stigma, allocating resources (69), providing training opportunities, and acknowledging efforts to promote positive change within the organization.

Regularly evaluating the effectiveness of interventions to improve mental health literacy and reduce stigma among clinical nurses is crucial for identifying areas of improvement and refining strategies over time (70). Collecting feedback from nurses, monitoring key performance indicators, and conducting periodic assessments can help gauge the impact of interventions and inform future initiatives.

Furthermore, it’s noteworthy that while more knowledge about mental ill health doesn’t necessarily equate to increased stigma, studies have indicated a desire for more education and experiences related to mental ill health among participants (71). Therefore, broadening the scope and depth of mental health knowledge among nurses and teaching effective patient interaction strategies may help mitigate stigma. Additionally, nurses should be equipped with appropriate coping strategies for their mental health, as the well-being of healthcare professionals can significantly impact staff and patient safety.

## 5 Conclusion

This study highlights the intricate interplay between mental health knowledge, stigma, and social distance among clinical nurses in China. The findings reveal that stigma serves as a mediating factor influencing the relationship between knowledge and social distance. Nurses with higher levels of mental health knowledge exhibited lower stigma and greater willingness to engage with people with a mental health condition, underscoring the critical role of education in shaping attitudes and behaviors. From a theoretical perspective, this research enriches the understanding of stigma dynamics within the nursing profession by integrating the Knowledge-Attitude-Practice (KAP) model. Practically, the findings emphasize the urgent need for targeted educational interventions to address stigma and improve mental health literacy among nurses.

## 6 Limitations and prospects

Despite these contributions, the study has limitations. The use of a convenience sample from a single region may limit the generalizability of the findings. Additionally, the cross-sectional design restricts causal inferences, necessitating longitudinal studies to further explore the causal pathways between knowledge, stigma, and social distance.

Future research should expand the scope to include diverse cultural and geographical contexts and investigate the effectiveness of stigma-reduction interventions in clinical settings. By addressing these challenges, future studies can provide more comprehensive insights into fostering stigma-free environments in healthcare and enhancing the professional development of nurses in mental health care.

## Data Availability

The raw data supporting the conclusions of this article will be made available by the authors, without undue reservation.
